# ECG Ventricular Repolarization Dynamics during Exercise: Temporal Profile, Relation to Heart Rate Variability and Effects of Age and Physical Health

**DOI:** 10.3390/ijerph18189497

**Published:** 2021-09-09

**Authors:** Adrián Hernández-Vicente, David Hernando, Germán Vicente-Rodríguez, Raquel Bailón, Nuria Garatachea, Esther Pueyo

**Affiliations:** 1 Growth, Exercise, NUtrition and Development (GENUD) Research Group, University of Zaragoza, 50009 Zaragoza, Spain; gervicen@unizar.es (G.V.-R.); nugarata@unizar.es (N.G.); 2 Department of Physiatry and Nursing, Faculty of Health and Sport Science (FCSD), University of Zaragoza, 22002 Huesca, Spain; 3 Red española de Investigación en Ejercicio Físico y Salud en Poblaciones Especiales (EXERNET), 50009 Zaragoza, Spain; 4 Biomedical Signal Interpretation and Computational Simulation (BSICoS), Aragón Institute for Engineering Research (I3A), IIS Aragón, University of Zaragoza, 50018 Zaragoza, Spain; dhernand@unizar.es (D.H.); rbailon@unizar.es (R.B.); epueyo@unizar.es (E.P.); 5 CIBER de Bioingeniería, Biomateriales y Nanomedicina (CIBER-BBN), 28029 Madrid, Spain; 6 Centro de Investigación Biomédica en Red de Fisiopatología de la Obesidad y Nutrición (CIBER-Obn), 28029 Madrid, Spain; 7 Instituto Agroalimentario de Aragón -IA2- CITA-Universidad de Zaragoza, 50013 Zaragoza, Spain

**Keywords:** electrocardiography, ventricular repolarization, time-frequency analysis, sympathetic nervous system, periodic repolarization dynamics, heart rate variability, exercise test, cluster analysis

## Abstract

Periodic repolarization dynamics (PRD) is a novel electrocardiographic marker of cardiac repolarization instability with powerful risk stratification capacity for total mortality and sudden cardiac death. Here, we use a time-frequency analysis approach to continuously quantify PRD at rest and during exercise, assess its dependence on heart rate variability (HRV) and characterize the effects of age (young adults/middle-aged adults/older adults), body mass index (non-overweight/overweight) and cardiorespiratory fitness level (fit/unfit). Sixty-six male volunteers performed an exercise test. RR and dT variabilities (RRV, dTV), as well as the fraction of dT variability unrelated to RR variability, were computed based on time-frequency representations. The instantaneous LF power of dT (P_dTV_), representing the same concept as PRD, and of its RRV-unrelated component (P_dTVuRRV_) were quantified. dT angle was found to mostly oscillate in the LF band. Overall, 50–70% of P_dTV_ was linearly unrelated to RRV. The onset of exercise caused a sudden increase in P_dTV_ and P_dTVuRRV_, which returned to pre-exercise levels during recovery. Clustering analysis identified a group of overweight and unfit individuals with significantly higher P_dTV_ and P_dTVuRRV_ values at rest than the rest of the population. Our findings shed new light on the temporal profile of PRD during exercise, its relationship to HRV and the differences in PRD between subjects according to phenotypic characteristics.

## 1. Introduction

Sudden cardiac death is responsible for 15–20% of all deaths in Western societies [1]. It is strongly associated with, and can be caused by, ventricular arrhythmias. Although rare, when an athlete’s life is claimed by sudden cardiac death, the impact on society is very high. Nevertheless, the absolute number of cases in athletes is not higher than in the general population, but intense exercise appears to increase the risk of sudden cardiac death in individuals harboring certain cardiac conditions [2]. Considering that less than 5% of people with an out-of-hospital cardiac arrest survive, the search for reliable markers able to identify athletes and non-athletes at high arrhythmic risk is urgently needed. This would help in the election of a cost-effective treatment, such as antiarrhythmic drugs, prophylactic implantation of a cardioverter defibrillator or catheter ablation [3]. Among the variety of non-invasive methods proposed in the literature to assess arrhythmic risk, methods can be found that quantify heart rate variability (HRV), baroreflex sensitivity or ventricular repolarization characteristics, such as the QT interval duration and hysteresis, T-wave alternans, T-peak-to-end/RR interval curvature or T-wave morphology restitution [4,5,6,7,8].

Sympathetic nervous system (SNS) hyperactivity has been shown to increase triggered activity and enhance dispersion of ventricular repolarization under different clinical conditions, thus contributing to accentuate the vulnerability to fatal ventricular arrhythmias and sudden cardiac death [9]. Recent studies have proposed an electrocardiogram (ECG)-based risk predictor, which has been suggested to reflect sympathetic effects on ventricular myocardium [10]. This marker, called periodic repolarization dynamics (PRD), quantifies the magnitude of low-frequency (LF) oscillations (≤0.1 Hz) in the angle dT between T-wave vectors of consecutive heart beats [10]. Elevated PRD measured at rest has been shown to be a strong predictor of all-cause mortality, cardiac mortality or sudden cardiac death in patients with acute and chronic myocardial infarction and in patients with chronic heart failure [10,11,12,13,14]. The stratification capacity of PRD has been shown to be independent of that of other clinical and ECG variables including left ventricular ejection fraction, HRV, diabetes mellitus and Global Registry of Acute Coronary Events score in myocardial infarction populations and New York Heart Association class in chronic heart failure populations [13,14]. In addition, PRD has been shown to predict mortality reduction associated with prophylactic implantation of defibrillators in cardiomyopathy patients and could thus help guide treatment decisions [15].

The physiological mechanisms underlying the genesis of PRD are yet to be fully described, particularly regarding the direct involvement of sympathetic oscillatory activity on regulation of the ventricular myocardium [9]. The importance of LF oscillations in providing information potentially related to sympathetic neural activity has been described through different markers obtained from the T-wave vector, sympathetic nerve activity recordings, HRV, action potentials or systolic arterial blood pressure [9]. Specifically regarding PRD, clinical and experimental studies have so far provided evidence that it is enhanced by sympathetic activation, induced by either tilt table test or exercise, and suppressed by pharmacological β-adrenergic blockade [10,16]. PRD has been verified to occur independently of respiratory activity in volume-controlled ventilated swine [10] and in humans when comparing respiratory rates of 10 and 20/min with constant minute ventilation [17]. Moreover, PRD has been confirmed not to be an epiphenomenon of HR and HRV, as substantiated by the fact that fixed atrial pacing exerts modest effects on PRD despite fully abolishing HRV and despite varying HR at fixed values in a large range [10,18]. This has been later supported by studies using an incremental exercise test, which have described low correlation between PRD and HR or HRV [16]. Nevertheless, there is yet limited information on how PRD varies continuously with time so that a full characterization of its temporal profile following physiological sympathoexcitatory interventions can be established. In addition, no study has so far provided quantifications of the PRD fractions related and unrelated to HRV and on the variation of these two fractions with time in response to sympathetic provocations. This is of interest, both in a general population but also in subpopulations stratified by certain phenotypic characteristics.

The aim of this study is to continuously quantify PRD at rest and during exercise, assess its dependence on heart rate variability (HRV) and characterize the effects of age, body mass index (BMI) and cardiorespiratory fitness level. For this purpose, we use a time-varying nonparametric methodology to evaluate the instantaneous power of LF oscillations in the dT angle, representing the same concept as PRD, and we ascertain the part of it that is unrelated to HRV and could thus reflect direct sympathetic effects on the ventricular myocardium. Next, we characterize how the LF power of dT and its HRV-unrelated portion change in response to incremental exercise in a population of subjects with highly varied age, BMI and cardiorespiratory fitness level. By clustering analysis, we identify groups of individuals with similar phenotypic characteristics and we assess differences in the magnitude of their LF oscillatory ventricular activities that could offer hints on the relationship between elevated PRD and cardiovascular risk.

## 2. Materials and Methods

### 2.1. Participants

Recruitment posters were distributed in public establishments, such as sports centers, hospitals and universities. Sixty-six males agreed to participate in the study. The sample consisted of three age groups: young adults from 20 to 30 years (N = 24), middle-aged adults from 40 to 50 years (N = 21) and older adults from 60 to 70 years (N = 21). Exclusion criteria included the following: subjects going through an acute disease, being on cardiac medication, suffering from heart diseases (e.g., atrial fibrillation or heart failure) or presenting any clinical contraindication for the practice of physical exercise. The descriptive characteristics of the three age groups are shown in Table 1. The study was conducted according to the guidelines of the Declaration of Helsinki and was approved by the ethical committee for clinical research of Aragón (ID of the approval: PI17/0409). After detailed explanation of potential risks, informed consent was obtained from all subjects involved in the study.

### 2.2. Procedure

All subjects performed an exercise test during a session at the laboratory between 16:00–20:00 h. Before the session, volunteers were asked to follow some guidelines: (1) refrain from doing heavy exercise the day before the test; (2) get enough sleep (6–8 h) the night before the test; (3) avoid substances such as alcohol, tobacco or stimulants (theine, taurine, caffeine, etc.) in the 8 h preceding the test; (4) do not eat for 3 h prior to the test; (5) ensure being well hydrated; and (6) wear comfortable clothing. Volunteers were prepared by using a razor to shave any hair from the electrode sites and by cleaning their skin with alcohol and gauze. To place the ECG electrodes, the manufacturer’s instructions were followed (H12 +, Mortara Instrument; Milwaukee, WI, USA).

The test was conducted in an environmentally controlled room (22–23 °C, 40–60% humidity) and was divided into 3 consecutive segments: resting (S_REST_), cycling (S_CY_) and recovery (S_REC_). During S_REST_, participants were monitored while seated at rest for 5 min, without moving or talking. A 3-min period was set to change from being seated in the chair during S_REST_ to being seated in the cycle ergometer during S_CY_. During this 3-min period, the volunteer rode the cycle-ergometer (Ergoselect 200 K, Ergoline; Bitz, Germany) at 50 W workload and chose a cadence that was maintained during the whole test. S_CY_ was a submaximal cycle-ergometer test divided into three stages lasting 5 min each. The workload was adjusted during each stage to 60, 70 and 80% of estimated maximum heart rate (HR_max_), with these stages denoted as S_CY60_, S_CY70_ and S_CY80_, respectively. HR_max_ was estimated for each subject by using HR_max_ = 208-0.7*age (years) to avoid a maximal exercise test [19]. Finally, during S_REC_, participants remained seated in the chair again for 5 min without moving or talking.

### 2.3. Data Recording

Volunteers self-reported their birth date, current medication and pathologies. Height was measured with a stadiometer (SECA 225; Hamburg, Germany) to the nearest 0.001 m, with participants standing and their heels, buttocks and scapula resting against a wall with the heels touching and forming a 45° angle and the head in the Frankfort’s plane. An electronic scale (SECA 861; Hamburg, Germany) was used to weight the subject to the nearest 0.1 kg, in underwear and after urination. BMI was calculated by dividing weight in kilograms by the square of height in meters. Based on World Health Organization standards, weight status was split into 2 groups: “non-overweight” (BMI < 25 kg·m^−2^) and “overweight” (BMI ≥ 25 kg·m^−2^) [20].

Submaximal exercise test is a safe and feasible method to estimate VO_2max_, showing good validity against maximal exercise tests (correlation coefficients from 0.69 to 0.98) [21]. Rather than commonly used tests with stages of short or variable duration, an ad hoc test with 5-min stages was defined to allow reliable estimation of the LF power of HRV and repolarization variability. This enabled assessment of cardiac response to increased sympathetic activity with each cycling stage [22]. Consequently, cardiorespiratory fitness was assessed using the approach of “Physical Work Capacity” (PWC) [23,24]. PWC was measured in watts during S_CY80_ of the submaximal cycle-ergometer test and was subsequently divided by the participant’s body weight (PWC_80%_ in W·kg^−1^). Alternatively to the use of fixed HR thresholds, this method incorporates the age-dependent decline of HR_max_ [23,24] and has previously been used as an objective assessment of cardiorespiratory fitness [25,26]. In each age group, cardiorespiratory fitness status was dichotomized: subjects above the W·kg^−1^ age group median were classified as “fit” and subjects below the age group median as “unfit”.

A twelve-lead high-resolution (1000 Hz) Holter recorder (H12+, Mortara Instrument; Milwaukee, WI, USA) was used to record the ECG.

### 2.4. Data Analysis and Processing

QRS detection and ECG wave delineation were performed by using a wavelet-based single-lead automatic system [27]. The detection and delineation annotations from each lead were combined by using rules to obtain multi-lead ECG delineation marks [27] and additional updates were applied to account for the high levels of noise during stress testing [28]. From these annotations, the RR time series (measured from one QRS complex to the next one) was extracted. In addition, the onset and end of the T-waves (T_on_ and T_end_, respectively) were obtained.

The time series of angles between consecutive T-wave vectors, denoted as dT series, was obtained as described in [29], which uses a method updated from the original one proposed in [10]. First, the orthogonal leads X, Y, Z were obtained from the 12-lead ECG using the inverse Dower matrix [30]. Each T-wave was delimited based on the T_on_ and T_end_ time points identified as described previously and an average T-wave vector was calculated for each wave. The angle dT between two consecutive T-waves was calculated by the dot product of each pair of consecutive average T-wave vectors.

Outlier values in both RR and dT time series were detected and corrected as described next. First, a 30-th order median filter was applied over the times series of absolute differences between successive intervals. Outliers were identified if their absolute difference was above 5 times the corresponding value in the median filtered series. These outlier values were replaced with the mean of their adjacent values.

RR variability (RRV), dT variability (dTV) and dT variability unrelated to RR variability (dTVuRRV) were computed based on time-frequency representations following previously developed approaches [31], as described next. First, a highpass filter with a cut-off frequency of 0.03 Hz was applied to both RR and dT series. To obtain the time-frequency (TF) representations, Cohen’s class distributions were used with temporal and spectral resolutions of 11.7 s and 0.039 Hz, respectively. TF representations of the dTV and RRV series, as well as the TF coherence between dTV and RRV series, were obtained and denoted as $S_{dTV}$(*t*,*f*), $S_{RRV}$(*t*,*f*) and $\gamma_{dTV,RRV}$(*t*,*f*), respectively. The TF spectrum of dTV was decomposed into two separate spectra, which allowed characterizing the part of dTV linearly related to RRV (dTVrRRV) and the part of dTV unrelated to RRV (dTVuRRV). The TF spectrum of dTVuRRV was calculated as:

(1) $$S_{dTVuRRV}\left(t,f\right)=\left(1-{\left|\gamma_{dTV,RRV}\left(t,f\right)\right|}^{2}\right)S_{dTV}\left(t,f\right)$$

The bias from the TF coherence estimators was estimated and corrected [31].

The instantaneous powers of LF oscillations for dTV, RRV, dTVuRRV and dTVrRRV series were calculated by integrating their TF distributions, $S_{dTV}$(*t*,*f*), $S_{RRV}$(*t*,*f*), $S_{dTVuRRV}$(*t*,*f*) and $S_{dTVrRRV}$(*t*,*f*) respectively, in the 0.03–0.15 Hz band, and denoted as $P_{dTV}$(*t*), $P_{RRV}$(*t*),$\;P_{dTVuRRV}$(*t*) and $P_{dTVrRRV}$(*t*). The normalized LF power of dTVuRRV was estimated as:

(2) $$P_{dTVuRRVn}\left(t\right)=\frac{P_{dTVuRRV}\left(t\right)}{P_{dTV}\left(t\right)}$$

From the instantaneous power series $P_{RRV}$(*t*),$\;P_{dTV}$(*t*), $P_{dTVuRRV}$(*t*), $P_{dTVrRRV}$(*t*), and $P_{dTVuRRVn}$(*t*), the indices used in the statistical analysis were obtained as the mean of the corresponding segment (S_REST_, S_CY60_, S_CY70_, S_CY80_ and S_REC_) after removing the first 30 s of each of them.

### 2.5. Statistical Analysis

The normality of data was checked using the Kolmogorov–Smirnov test. Since the data distribution violated the assumption of normality necessary for the parametric tests and could not be corrected by commonly employed transformations, non-parametric analysis was conducted. Descriptive variables are presented as mean ± standard deviation and markers related to cardiac variability series are reported as median and interquartile range. Statistical analyses were performed using IBM SPSS (version 25; Chicago, IL, USA). The significance level was set at *p* ≤ 0.05.

Friedman’s two-way ANOVA, the non-parametric equivalent of one-way related analysis of variance ANOVA, was used to test for differences in variables between test segments, i.e., S_REST_, S_CY60_, S_CY70_, S_CY80_, and S_REC_. The Dunn–Bonferroni post hoc method was used for pairwise comparisons.

Cluster analysis was performed to identify groups of subjects with similar characteristics in terms of the following three variables of interest: age, BMI and cardiorespiratory fitness level (PWC_80%_). Following the methodology of previous studies [32,33], two types of cluster analyses were combined: hierarchical clustering (Ward’s method) and k-means clustering. First, individual and multivariate outliers (according to Mahalanobis distance) were detected to reduce the sensitivity of the Ward’s method to outliers. Second, hierarchical cluster analysis was used, as the number of clusters in the data was unknown beforehand. Examination of dendrograms showed that a two-cluster solution produced good differentiation between groups. Finally, k-means cluster was performed with two possible solutions. Compared to hierarchical methods, k-means cluster analysis is considered less sensitive to outliers and has been found to result in greater within-cluster homogeneity and between-cluster heterogeneity [32].

A Kruskal–Wallis test (non-parametric equivalent of one-way independent ANOVA) with Bonferroni correction was performed to assess differences in variables between the three age groups, i.e., young adults, middle-aged adults and older adults. The Dunn–Bonferroni post hoc method was used for pairwise comparisons. To evaluate the magnitude of the differences, ES was calculated as: $ES=H/(\left(n^{2}-1\right)/\left(n+1\right))$, where *H* stands for the Kruskal–Wallis test statistic and *n* is the total number of observations [34].

The Mann–Whitney *U*-test, the non-parametric equivalent of the unpaired samples t-test, was used to determine differences in variables between dichotomous groups i.e., BMI (non-overweight/overweight), PWC_80%_ (fit/unfit) and clusters (CLUSTER A/B). The magnitude of the difference was calculated by determining the effect size (ES): $ES=Z/\sqrt{n}$ where *Z* represents the Z-score for the Mann–Whitney *U*-test and *n* is the total number of observations [34]. The difference was considered small when ES < 0.2, small to medium when ES = 0.2–0.5, medium to large when ES = 0.5–0.8 and large when ES > 0.8 [35].

## 3. Results

### 3.1. LF Oscillations of dT in Response to Exercise and Relation to HRV

**Figure 1:**
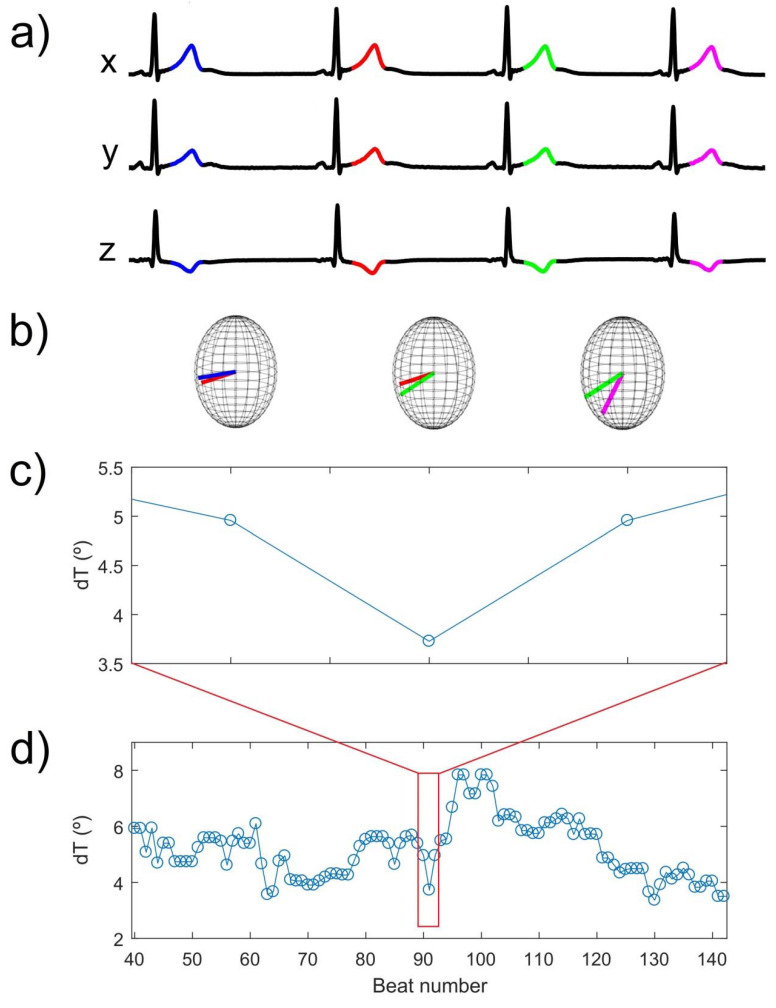
Computation of dT from an ECG in the Frank lead configuration. (**a**) T-waves from four consecutive heart beats. (**b**) Pairs of T-wave vectors from consecutive beats are illustrated in three-dimensional spheres. (**c**) Angle dT between the consecutive T-wave vectors shown in panel b. (**d**) Time series of dT for a piece of an ECG recording.

Figure 1 shows the concept of dT, with representation of the orthogonal leads X, Y, and Z derived from the twelve standard leads (Figure 1a) and pairs of T-wave vectors corresponding to consecutive beats (Figure 1b). The time series of dT shows the time course of the angles between pairs of T-wave vectors (Figure 1d, zoomed version in Figure 1c).

**Figure 2:**
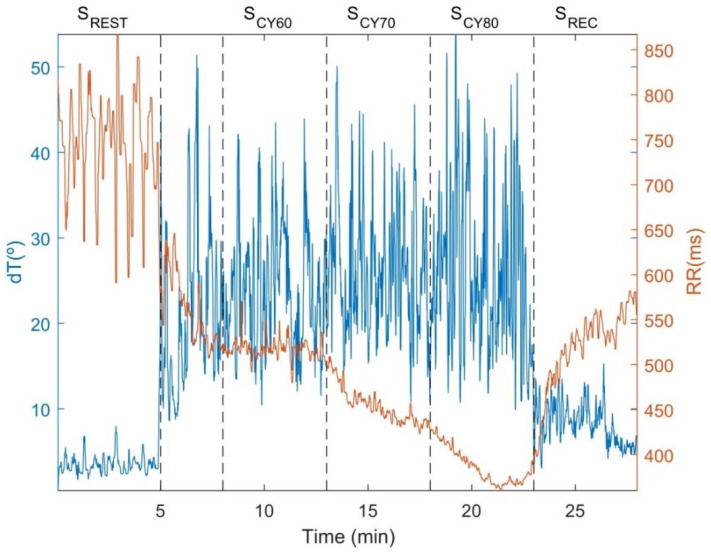
Example of the dT angle and RR interval time series for one subject throughout the entire test. Dotted lines separate the different test segments: resting (S_REST_), cycling (S_CY_) and recovery (S_REC_). S_CY_ was divided into three stages corresponding to 60, 70 and 80% of estimated HR_max_, denoted as S_CY60_, S_CY70_ and S_CY80_, respectively.

**Figure 3:**
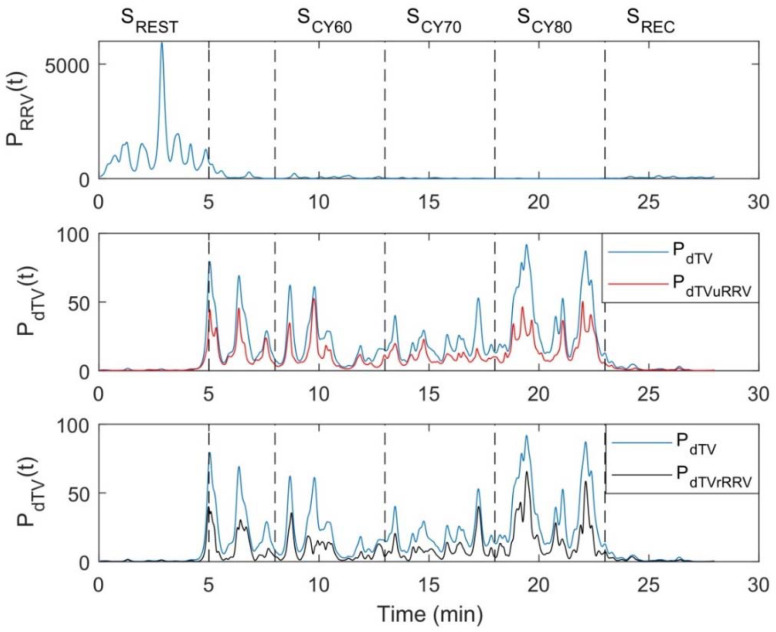
Example of the instantaneous power of LF oscillations for: RR variability (P_RRV_), dT variability (P_dTV_), dTV unrelated to RRV (P_dTVuRRV_) and dTV related to RRV (P_dTVrRRV_) obtained for the same subject as in Figure 2. Dotted lines separate the different test segments: resting (S_REST_), cycling (S_CY_) and recovery (S_REC_). S_CY_ was divided into three stages corresponding to 60, 70 and 80% of estimated HR_max_, denoted as S_CY60_, S_CY70_ and S_CY80_, respectively.

**Figure 4:**
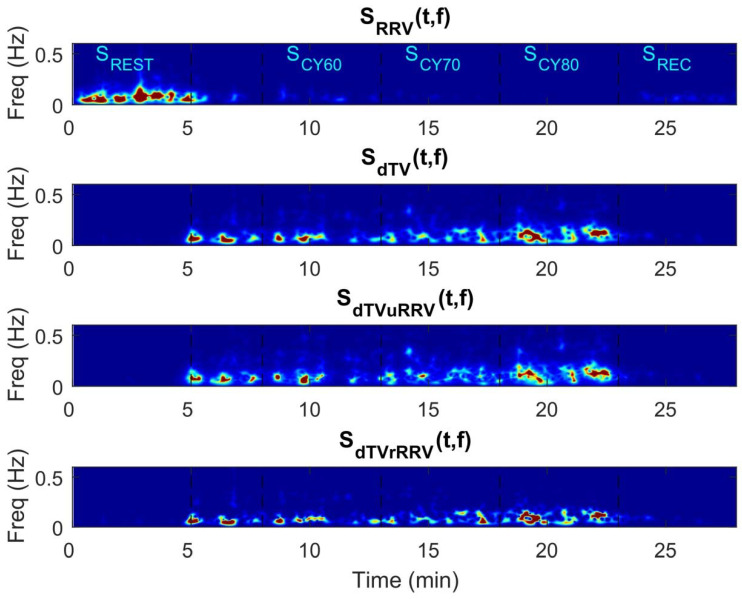
Example of the time-frequency distribution for RR variability (RRV), dT variability (dTV), dTV unrelated to RRV (P_dTVuRRV_) and dTV related to RRV (P_dTVrRRV_) obtained for the same subject as in Figure 2. Dotted lines separate the different test segments: resting (S_REST_), cycling (S_CY_) and recovery (S_REC_). S_CY_ was divided into three stages corresponding to 60, 70 and 80% of estimated HR_max_, denoted as S_CY60_, S_CY70_ and S_CY80_, respectively.

Figure 2, Figure 3 and Figure 4 illustrate the temporal evolution of the RR and dT indices and their variabilities throughout the test, including rest, cycling and recovery. Figure 2 shows an example of dT and RR time series from a subject of the study population, from which it can be observed that there are decreases in both RR and its variability during exercise, concomitant to increases in dT and its variability. The corresponding temporal evolution of the instantaneous power of LF oscillations for RRV (P_RRV_, i.e., LF component of RRV), dTV (P_dTV_, representing the PRD concept), dTV unrelated to RRV (P_dTVuRRV_, i.e., the fraction of PRD unrelated to the LF component of RRV) and dTV related to RRV (P_dTVrRRV_) is displayed in Figure 3. The relevant contribution to dTV of both its RRV-related and RRV-unrelated components can be clearly appreciated, with the two of them showing remarkable increases during exercise. The time-frequency distributions of RRV, dTV, dTVuRRV and dTVrRRV are depicted in Figure 4. Oscillations in dT are very notable in magnitude during exercise and are mostly concentrated in the LF band. Although a portion of dTV is related to RRV, there is an important fraction of it that provides information additional to RRV.

**Figure 5:**
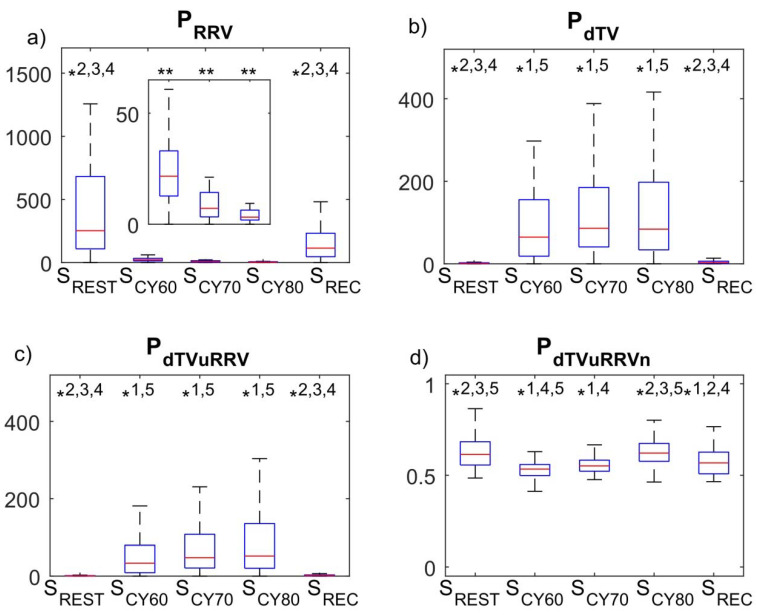
Box plots representing the distributions of P_RRV_, P_dTV_, P_dTVuRRV_ and P_dTVuRRVn_ (N = 66) over the study population (N = 66) for the different test segments: resting (S_REST_), cycling (S_CY_) and recovery (S_REC_). S_CY_ was divided into three stages corresponding to 60, 70 and 80% of estimated HR_max_, denoted as S_CY60_, S_CY70_ and S_CY80_. (**a**) LF oscillations of RR variability (P_RRV_), with the inset showing the three S_CY_ stages. (**b**) LF oscillations of dT variability (P_dTV_). (**c**) P_dTV_ unrelated to P_RRV_ (P_dTVuRRV_). (**d**) Normalized P_dTV_ unrelated to P_RRV_ (P_dTVuRRVn_). * = Significant differences between test segments (*p* ≤ 0.05, Friedman’s ANOVA): * 1 = Different to S_REST_; * 2 = Different to S_CY60_; * 3 = Different to S_CY70_; * 4= Different to S_CY80;_ * 5 = Different to S_REC;_ ** = Different to all.

Figure 5 shows box plots representing the distributions of P_RRV_, P_dTV_, P_dTVuRRV_ and P_dTVuRRVn_ over the study population (N = 66) for the different test segments. Regarding P_RRV_ shown in Panel 5.a, the beginning of exercise elicits a drop in the LF oscillations of RRV (*p* ≤ 0.001), followed by a progressive decrease with each increment in exercise intensity (all *p* ≤ 0.05), upon which P_RRV_ returns (*p* ≤ 0.001) to pre-exercise levels in the recovery segment. The P_dTV_ profile in Panel 5.b shows that exercise onset causes a sudden increase well above the resting level (*p* ≤ 0.001), followed by a variable behavior among subjects during exercise (note the wide boxes and whiskers in S_CY60_, S_CY70_ and S_CY80_), with a subsequent decrease in P_dTV_ corresponding to the recovery segment (*p* ≤ 0.001), at the end of which values similar to rest are attained. From Panel 5.c, it can be seen that P_dTVuRRV_ has a similar pattern to P_dTV_, in this case with a more remarkable tendency to increase in median with exercise intensity, although this increase is not statistically significant due to the wide distribution of values at S_CY60_, S_CY70_ and S_CY80_. P_dTVrRRV_ also has a similar pattern to P_dTV_, but without any observable tendency to increase in median with exercise intensity as seen in P_dTVuRRV_. Finally, Panel 5.d shows the normalized P_dTVuRRV_, i.e., P_dTVuRRVn_, with values at S_CY60_ and S_CY70_ being significantly lower than at S_CY80_ (*p* ≤ 0.001).

### 3.2. Effects of Age, BMI and Cardiorespiratory Fitness Level on LF Oscillations of dT

Table A1 shows averaged values of P_RRV_, P_dTV_, P_dTVuRRV_, P_dTVrRRV_ and P_dTVuRRVn_ for young, middle-aged and older adults across the different test segments. Significant reductions with age were found in P_RRV_ at S_REST_, S_CY60_ and S_REC_. Table A2 presents the comparison of the same indices according to BMI groups. P_dTV_ and P_dTVrRRV_ at S_REST_ were significantly higher in the overweight group, while P_dTVuRRVn_ at S_CY60_ and P_RRV_ at S_REC_ were significantly higher in the non-overweight group. The corresponding comparison according to cardiorespiratory fitness levels is shown in Table A3. Only P_dTVuRRVn_ at the highest exercise intensity, i.e., S_CY80_, was significantly higher in the more fit individuals.

### 3.3. Identification of Individuals with Elevated LF Oscillations of dT

Table 2 shows the descriptive characteristics of the two cluster groups, which were described as CLUSTER A “non-overweight and fit” (normal BMI and high PWC_80%_), and CLUSTER B “overweight and unfit” (high BMI and low PWC_80%_).

Table 3 shows averaged values of P_RRV_, P_dTV_, P_dTVuRRV_, P_dTVrRRV_ and P_dTVuRRVn_ for the two cluster groups. At S_REST_, P_dTV_, P_dTVuRRV_ and P_dTVrRRV_ were significantly higher for CLUSTER B, while P_RRV_ was significantly higher for CLUSTER A. During the other test segments, results were not significantly different between groups, except for P_RRV_ at S_REC_.

**Figure 6:**
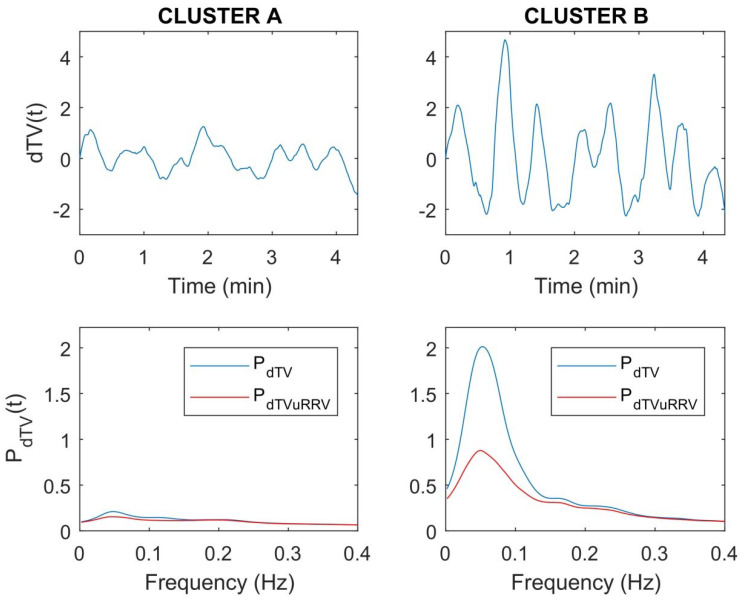
Examples of dTV, P_dTV_ and P_dTVuRRV_ at rest obtained for two subjects that are representative of each of the two clusters. A and B, described in the text.

Figure 6 shows examples of dT time series at rest for two subjects that are representative of each of the two clusters. The more pronounced LF oscillations in dT for the subject belonging to CLUSTER B can be clearly appreciated (top panels). This is manifested in higher instantaneous P_dTV_, with the RRV-unrelated fraction of it, P_dTVuRRV_, remaining higher too (bottom panels).

## 4. Discussion

By using time-frequency methods, we confirmed that the dT angle between consecutive T-wave vectors mainly oscillates in the LF band and, for the first time, we showed that its variability can be decomposed into two components with relevant contributions, one related to RRV and the other one unrelated to it. As an advantage of our methods over other methods quantifying LF oscillations of dT, we could characterize the temporal profile of dTV and of its RRV-related and unrelated components during an exercise test. In line with previous findings, we observed a significant exercise-induced increase in the instantaneous LF power of dTV, P_dTV_, with respect to rest and recovery, which we proved to be accompanied by gradual increases in its RRV-unrelated component, P_dTVuRRV_, but not in its RRV-related one, P_dTVrRRV_, in response to incremental exercise. The temporal profile of P_dTV_ and P_dTVuRRV_ as a function of exercise intensity was highly inter-individual. Importantly, our study provides first evidence on the behavior of P_dTV_ as a function of age, BMI and cardiorespiratory fitness level, both when these variables are analyzed individually and in combination. We showed that, at rest but not along incremental exercise, P_dTV_, P_dTVuRRV_ and P_dTVrRRV_ were significantly elevated in a group of overweight and unfit individuals, while no clear relationship with age could be established.

### 4.1. dT Mainly Oscillates in the LF Band, Being Not Completely Unrelated to HRV

To the best of our knowledge, this study is the first one using time-frequency methods to evaluate the frequency components of dTV and RRV during rest, exercise and recovery. The results of our ECG analysis corroborate that dT oscillations are mainly contained in the LF band and their magnitude is enhanced in response to exercise-induced sympathetic stimulation [10,16,18]. These results agree well with previous reports showing enhancement of PRD, which represents the same concept as P_dTV_, subsequent to tilt table test and to mild exercise as well as decrease following β-adrenergic blockade [10]. On top of clinical and experimental studies assessing LF oscillations in ventricular repolarization from the surface ECG [13,14], these oscillations have additionally been demonstrated by in vivo studies at the level of ventricular electrograms and action potentials, which have characterized the LF oscillatory pattern [36,37,38,39], its magnification by sympathetic provocations [37] and its reduction following β-adrenergic blockade [38]. In silico studies have suggested that synergistic β-adrenergic stimulation and mechanical stretch could contribute to explain the LF oscillatory pattern of ventricular repolarization [40,41]. Although further research is needed to mechanistically link LF oscillations in ventricular repolarization to LF rhythmic discharge of sympathetic neurons [9], our study, together with all cited evidences from cell to whole-body levels, provide indirect support to the involvement of the sympathetic nervous system in the generation of the observed oscillatory behavior.

An important aspect that could render repolarization risk markers, such as PRD, inaccurate in representing the sympathetic effect on ventricular repolarization is their dependence on HR. Here, we show that P_dTV_ has an RRV-unrelated fraction accounting for 50–70% of it and an RRV-related one accounting for the remaining 30–50%. For PRD to be more meaningful from a clinical point of view, its RRV-unrelated fraction could be analyzed, as it could more closely reflect ventricular repolarization instabilities occurring under excessive sympathetic activity that may increase susceptibility to ventricular arrhythmias and sudden cardiac death. Prior studies investigating PRD have assessed its modulation by HR by evaluating the response to physiological interventions, such as hyperventilation or incremental exercise, and have established the independence of dT and PRD with respect to HR and HRV by reporting a non-significant correlation [16,17]. Other studies have determined that PRD is not an epiphenomenon of HRV by proving that it presents small (25% in mean) changes following fixed atrial pacing to abolish HRV [10]. Here, we provide instantaneous quantification of the percentages of P_dTV_ that are related and unrelated to RRV at any time instant during rest, exercise and recovery. This quantification could prove useful to assess whether increases in RRV-unrelated oscillations of dT could be more sensitive in predicting impeding ventricular arrhythmias than the combined RRV-related and unrelated oscillations measured through PRD. Previous studies in the literature have confirmed the value of specifically measuring RRV-unrelated oscillations of other ventricular repolarization markers such as the QT interval. In a recent investigation, the LF power of QT variability (QTV) unrelated to RRV, but not of the full QTV, was able to identify patients with coronary artery disease (CAD) from the first phases of a stress test [42]. In [43], QTV was quantified at given HRV levels and it was reported to be greater in heart failure patients with spontaneous ventricular tachycardia than in normal heart subjects, with inter-group QTV differences being further amplified in response to atrial pacing (i.e., in the absence of HRV). These evidences on the existence and value of mechanisms additional to RRV-dependent effects on LF oscillations of ventricular repolarization provide new avenues for the development of arrhythmic risk markers with improved stratification capacity by refinement of PRD, as suggested in this study.

### 4.2. Incremental Exercise Enhances LF Oscillations of dT, with the Temporal Oscillatory Profile Being Highly Inter-Individual

While the pattern of change in RRV along a full exercise test has been extensively described in the literature, the pattern of dTV remains less well characterized. In line with previous reports [44], we describe a sudden drop in P_RRV_ with the beginning of exercise, followed by a more gradual decay as exercise intensity increases and a return to resting P_RRV_ levels during the recovery segment. Regarding dT and P_dTV_, only two studies have provided an in-depth description of the pattern of change during the exercise test [16,18]. In agreement with these two studies, we show that, with the start of the exercise and the elevation in the sympathetic activity, there is an increase in dT and P_dTV_, with such an increase being sustained along the different exercise intensities in mean over the analyzed population. In some individuals, P_dTV_ is magnified by a factor above 200 at maximum exercise intensity. In accordance to previous works, we show a decrease in P_dTV_ towards pre-exercise values during recovery [16,18].

The specific characteristics of the dT and P_dTV_ profiles along an exercise test vary across studies depending on the design of the exercise protocol. Hamm et al. used a step-wise incremental protocol and described that dT increased concordantly to HR until reaching the lactate anaerobic threshold and then started to decline discordantly to HR [18]. Milagro et al. considered a more exigent ramp protocol and did not observe such a transient drop in dT but reported a three-phase profile of dT and P_dTV_ during exercise. This profile consisted of an initial rapid rise and plateau-like behavior at light-intensity exercise, followed by a slight increase around the point when P_RRV_ reached its minimum and a final sudden increase after reaching the second ventilatory threshold [16]. Here, we find a tendency for P_dTV_ to increase with exercise intensity, even if not reaching statistical significance possibly due to the fact that, at our analyzed intensities, not all subjects reached the second ventilatory threshold (around 80–90% of HR_max_) after which dT and P_dTV_ would be expected to grow remarkably [16,45].

On top of characterizing the P_dTV_ profile, we provide the profiles of its RRV-related and unrelated fractions, not investigated so far in previous studies. While the RRV-unrelated fraction, P_dTVuRRV_, presents a similar pattern to P_dTV_, with an even more marked increment in relation to exercise intensity, this was not the case of the RRV-related fraction, P_dTVrRRV_, which did not show an increasing tendency with exercise intensity in mean over subjects. These results support the observation that the RRV-unrelated part of PRD could better reflect sympathetic effects on ventricular repolarization, with increased repolarization lability levels accompanying increased sympathetic activity [46]. Other studies in the literature have investigated the profile of repolarization variability and its RRV-unrelated component during exercise by analysis of QTV. In [42], the RRV-unrelated fraction of QTV is shown to be increased with exercise and to represent nearly 80% of all QTV at maximum exercise intensity. While this is true for both non-CAD and CAD patients, significant differences between these two groups are appreciated only at the first phases of the stress test and only for the RRV-unrelated fraction of QTV, which highlights the relevance of using methods able to separate the two repolarization variability components and to monitor them over the course of time, as proposed in this study. In addition, the time course of the LF power of the two QTV components has been investigated in response to maneuvers that shift the sympathovagal balance towards more sympathetic predominance, such as the tilt table test [31,47]. The unrelated component, but not the related one, increases significantly along the tilt test, again confirming the importance of the time-varying methodologies used in our study for characterization of LF oscillations of repolarization unrelated to RRV.

### 4.3. LF Oscillations of dT Are Significantly Elevated in a Group of Overweight and Unfit Individuals

The measurements of cardiac variability quantified in our study have been compared between groups stratified by age, BMI and cardiorespiratory fitness level. In accordance to the literature [48], we show that age is associated with a reduction in P_RRV_ at rest, light-intensity exercise and recovery. Additionally, we analyze, for the first time, the relationship between age and P_dTV_, P_dTVuRRV_ and P_dTVrRRV_ and we describe no significant differences between age groups. Similarly, when comparing according to BMI or cardiorespiratory fitness level individually, most variables did not show differences between groups either, with only P_dTV_ and P_dTVrRRV_ at rest being significantly higher in the overweight group.

Next, we performed cluster analysis to identify subjects with common phenotypic characteristics. CLUSTER A, composed of “non-overweight and fit” individuals, presents higher P_RRV_ at rest than CLUSTER B comprising “overweight and unfit” individuals, which is congruent with studies associating higher HRV with better health and lower HRV with poorer prognosis in different clinical conditions [49]. In addition, CLUSTER B shows higher P_dTV_, P_dTVuRRV_ and P_dTVrRRV_ at rest, which agrees with investigations linking elevated resting P_dTV_ with higher cardiovascular risk (Rizas et al. 2014). Indeed, PRD has been shown to be a strong predictor of all-cause mortality, cardiac mortality and sudden cardiac death in different patient populations [10,11,12,13,14]. It should be noted that the way to calculate P_dTV_ in the present study is not the same as in some of the aforementioned clinical studies and, thus, our reported P_dTV_ values should not be compared with the PRD threshold set in those studies for mortality prediction. Future work in larger study populations should confirm whether P_dTV_ (equivalent to PRD) and its RRV-unrelated fraction, P_dTVuRRV_, can be used as tools to measure the chronic effects of age, BMI or fitness on sympathetically-modulated ventricular repolarization and how this could be related to increased cardiac and arrhythmic risk.

As an observation from our research, we could not find significant differences between clusters A and B in terms of LF oscillations of cardiac activity during exercise. Our initial hypothesis was that exercise would accentuate potential resting differences between individuals with distinct phenotypes. However, the temporal profile of P_dTV_ presents high inter-individual variability even among subjects of the same cluster, which results in a large standard deviation of the P_dTV_ measures. In terms of the median of P_dTV_ and its RRV-related and unrelated components (see Table 3), CLUSTER A shows an increasing trend with exercise intensity, whereas the opposite behavior is observed in CLUSTER B. Particularly for CLUSTER A, we show a marked increase in P_dTV_ from S_CY70_ to S_CY80_, which matches the findings by Milagro et al., who reported a sudden increase around the second ventilatory threshold in trained subjects [16]. Our observed differences between the two clusters could potentially be a reflection of differences in the sympathetic modulation of ventricular activity with exercise, with the profile reported for subjects of CLUSTER A being representative of a better health status.

### 4.4. Strengths, Limitations and Future Research

A key strength of the present study is the in-depth analysis of PRD (quantified through P_dTV_) using a time-frequency approach to track the frequency components of the dT time series and of their portions related and unrelated to RRV. Second, previous studies describing PRD patterns during exercise have been carried out in groups of 20 young lean volunteers and, in some cases, all of them being physically fit [16,18]. In contrast, our study population is larger, comprises volunteers of ages spanning from 20 to 70 years old and is much more heterogeneous in terms of weight and physical fitness, thus being more representative of the general population. Third, cluster analysis is used to evaluate the extent to which LF oscillations of HR and ventricular repolarization are modulated by the concurrence of phenotype characteristics, such as age, BMI and cardiorespiratory fitness levels. This perspective is particularly relevant considering that, by 2050, 1 out of 6 people in the world will be an older adult [50] and advanced age has been associated with changes in body composition and reduced cardiorespiratory fitness [51,52]. Last but not least, all the measurements and signal recordings of this study are performed in the laboratory, under homogeneous conditions, thus enabling control of confounding factors and guaranteeing the reproducibility of the study.

On the other hand, some limitations of the study are to be acknowledged. Although larger than in previous similar studies, the sample size is still relatively small. In future research, larger, more representative samples would allow confirming the findings of the present study regarding the temporal profile of PRD during incremental exercise, with dissection of the portion attributable to HR-dependent effects and the portion related to intrinsic autonomic modulation of the ventricular myocardium. Furthermore, it should be born in mind that a mesomorph subject may be overweight according to its BMI, so the results on ECG ventricular repolarization dynamics in these subjects should be critically interpreted taking this into account. As another limitation of our study, all the participants were Spanish white men. Further work should aim at applying the methodologies here reported onto other populations including women and other racial or ethnic groups.

In this paper, PRD is quantified during exercise and values are found to be two orders of magnitude higher than at rest. Previous studies have established thresholds for cardiac and arrhythmic risk stratification based on resting PRD measurements. Future studies could take the present work as a basis and measure PRD in clinical populations to assess the value of exercise-induced PRD increments for risk prediction. This, together with other more mechanistic investigations, could help elucidate the grounds underlying the predictive capacity of elevated PRD. Those grounds could involve not only a higher vulnerability of the myocardium to arrhythmogenic LF repolarization oscillations but a higher release of norepinephrine and arrhythmogenic co-transmitters due to larger neuronal synchronization, as proposed in [9]. Additionally, further research should confirm whether PRD and its RRV-unrelated component, both measured at rest and in response to exercise, could be useful to assess chronic effects of age, BMI and cardiorespiratory fitness level on ventricular activity and its relationship to cardiac risk, in general, and arrhythmic risk, in particular.

## 5. Conclusions

This study characterizes the frequency content along time of the dT angle between consecutive ECG T-wave vectors as a measure of repolarization instability. Oscillations in dT mostly occur in the low-frequency band and as much as 50–70% of them are unrelated to heart rate variability. The instantaneous LF power of dT, P_dTV_, increases by two orders of magnitude during an incremental exercise protocol as compared to values at rest and during recovery from exercise, although high inter-individual variability is observed in the temporal profiles of P_dTV_. By clustering analysis, we show that a group of overweight and unfit individuals presents significantly larger P_dTV_ values at rest, whereas no clear relationship with age is observed. Notwithstanding the limitations of the study, concerning sample size, BMI and sample characteristics, these findings extend our knowledge of periodic repolarization dynamics (PRD), a promising ECG risk marker, and set the stage for future studies to investigate exercise-induced heart rate-unrelated changes in PRD as a strategy to improve its prognostic cardiac and arrhythmic risk stratification capacity.

## Figures and Tables

**Table 1:** Descriptive characteristics of the three age groups.

Outcome	Young Adults (*n* = 24)	Middle-Aged Adults (*n* = 21)	Older Adults (*n* = 21)
Age (years)	25.41 ± 2.74	42.86 ± 3.06	63.82 ± 2.97
Height (m)	1.75 ± 0.06	1.77 ± 0.06	1.71 ± 0.05
Weight (kg)	71.81 ± 11.45	78.44 ± 10.49	76.53 ± 7.96
BMI (kg·m^−2^)	23.30 ± 2.86	25.09 ± 2.88	26.17 ± 2.82
“Overweight” (%)	20.8 (5)	47.6 (10)	61.9 (13)
PWC_80%_ (W·kg^−1^)	2.01 ± 0.61	2.02 ± 0.59	1.74 ± 0.59
“Unfit” (%)	50.0 (12)	52.4 (11)	52.4 (11)

Continuous variables are expressed as mean ± standard deviation. Dichotomous variables are expressed as percentage (number of subjects). BMI = Body mass index; PWC_80%_ = Physical Work Capacity at 80% of estimated HR_max_ (208-0.7*age in years) in watts per kg bodyweight.

**Table 2:** Descriptive characteristics of the two cluster groups.

Outcome	CLUSTER A (*n* = 31)	CLUSTER B (*n* = 35)	*p*	Effect Size
Age (years)	32.99 ± 11.48	52.22 ± 14.38	<0.001 *	0.580
Height (m)	175.37 ± 6.07	173.51 ± 6.20	0.203	0.157
Weight (kg)	69.36 ± 8.88	80.79 ± 8.57	<0.001 *	0.506
BMI (kg·m^−2^)	22.48 ± 1.87	26.82 ± 2.38	<0.001 *	0.757
*% of “overweight”*	*6.5 (2)*	*74.3 (26)*		
PWC_80%_ (W·kg^−1^)	2.33 ± 0.59	1.57 ± 0.33	<0.001 *	0.628
*% of “unfit”*	*32.3 (10)*	*68.6 (24)*		

Continuous variables are expressed as mean ± standard deviation. *Dichotomous variables* are expressed as *percentage (number of subjects)*. BMI = Body mass index; PWC_80%_ = Physical Work Capacity at 80% of estimated HR_max_ (208-0.7*age in years) in watts per kg bodyweight. Clusters were based on: age, BMI and cardiorespiratory fitness level (PWC_80%_). * = Significant differences between clusters (*p* ≤ 0.05, Mann–Whitney *U*-test).

**Table 3:** Comparison of P_RRV_, P_dTV_, P_dTVuRRV_, P_dTVrRRV_ and P_dTVuRRVn_ between cluster groups.

	Outcome	CLUSTER A (*n* = 31)	CLUSTER B (*n* = 35)	*p*	ES
S_REST_	P_RRV_ (e^−4^)	18.14 (6.31 to 38.94)	9.79 (3.65 to 17.54)	0.020 *	0.287
	P_dTV_	1.00 (0.41 to 1.87)	1.50 (0.82 to 2.96)	0.021 *	0.284
	P_dTVuRRV_	0.51 (0.22 to 1.23)	0.75 (0.49 to 1.56)	0.039 *	0.254
	P_dTVrRRV_	0.39 (0.23 to 0.79)	0.75 (0.37 to 1.43)	0.018 *	0.290
	P_dTVuRRVn_	0.62 (0.60 to 0.70)	0.62 (0.56 to 0.73)	0.743	0.040
S_CY60_	P_RRV_ (e^−4^)	1.40 (0.98 to 3.41)	1.64 (0.89 to 2.39)	0.400	0.104
	P_dTV_	63.42 (24.28 to 162.91)	99.78 (33.81 to 186.33)	0.289	0.130
	P_dTVuRRV_	32.34 (11.83 to 72.16)	53.95 (15.66 to 93.05)	0.295	0.129
	P_dTVrRRV_	30.84 (12.45 to 74.75)	48.50 (11.08 to 93.27)	0.415	0.100
	P_dTVuRRVn_	0.55 (0.52 to 0.58)	0.53 (0.50 to 0.56)	0.141	0.181
S_CY70_	P_RRV_ (e^−4^)	0.66 (0.32 to 1.21)	0.60 (0.33 to 1.15)	0.974	0.004
	P_dTV_	98.08 (54.45 to 186.19)	88.19 (69.84 to 243.71)	0.724	0.043
	P_dTVuRRV_	56.59 (29.10 to 106.70)	49.70 (30.67 to 126.59)	0.733	0.042
	P_dTVrRRV_	42.96 (21.45 to 79.49)	47.58 (22.86 to 83.74)	0.832	0.026
	P_dTVuRRVn_	0.56 (0.54 to 0.60)	0.55 (0.52 to 0.60)	0.272	0.135
S_CY80_	P_RRV_ (e^−4^)	0.24 (0.12 to 0.40)	0.22 (0.14 to 0.43)	0.729	0.043
	P_dTV_	136.21 (53.69 to 223.58)	79.23 (51.54 to 150.67)	0.352	0.115
	P_dTVuRRV_	83.28 (33.82 to 152.10)	48.80 (23.36 to 117.25)	0.372	0.110
	P_dTVrRRV_	47.41 (19.87 to 91.11)	30.82 (17.09 to 59.68)	0.256	0.140
	P_dTVuRRVn_	0.64 (0.61 to 0.70)	0.60 (0.57 to 0.67)	0.052	0.240
S_REC_	P_RRV_ (e^−4^)	9.03 (5.18 to 17.32)	5.29 (2.49 to 13.55)	0.036 *	0.259
	P_dTV_	2.36 (1.40 to 5.70)	3.82 (1.91 to 7.94)	0.079	0.216
	P_dTVuRRV_	1.21 (0.83 to 3.07)	2.35 (1.26 to 4.06)	0.060	0.232
	P_dTVrRRV_	1.14 (0.58 to 2.55)	1.90 (0.66 to 3.88)	0.250	0.142
	P_dTVuRRVn_	0.59 (0.53 to 0.64)	0.57 (0.53 to 0.63)	0.559	0.072

Values are expressed as median and interquartile range. Segments are based on the test phases: resting (S_REST_), cycling (S_CY_) and recovery (S_REC_). S_CY_ was divided in three stages at 60, 70 and 80% of estimated HR_max_, denoted as S_CY60_, S_CY70_ and S_CY80,_ respectively. P_RRV_ = LF oscillations for RR variability; P_dTV_ = LF oscillations for dT variability; P_dTVuRRV_ = P_dTV_ unrelated to P_RRV_; P_dTVrRRV_ = P_dTV_ related to P_RRV;_ P_dTVuRRVn_ = normalized P_dTVuRRV_. Clusters were based on: age, BMI and cardiorespiratory fitness level (PWC_80%_). ES = Effect size. * = Significant differences between groups (*p* ≤ 0.05, Mann–Whitney *U*-test).

## Data Availability

The datasets analyzed during the current study are available from the corresponding author on reasonable request.

## Appendix A

**Table A1:** Comparison of P_RRV_, P_dTV_, P_dTVuRRV_, P_dTVrRRV_ and P_dTVuRRVn_ between age groups at the different evaluated test segments.

	Outcome	Young Adults (*n* = 24)	Middle-Aged Adults (*n* = 21)	Older Adults (*n* = 21)	Main Effect	
					*p*	Effect Size
S_REST_	P_RRV_ (e^−4^)	23.17 (15.06 to 43.17) ^O^	11.96 (7.90 to 23.84) ^O^	3.65 (1.86 to 10.82) ^Y,M^	**<0.001 ***	0.333
	P_dTV_	1.31 (0.64 to 2.55)	1.33 (0.71 to 4.95)	0.92 (0.48 to 2.76)	0.666	0.012
	P_dTVuRRV_	0.80 (0.39 to 1.24)	0.64 (0.41 to 2.75)	0.61 (0.26 to 1.41)	0.712	0.010
	P_dTVrRRV_	0.54 (0.24 to 1.15)	0.69 (0.31 to 2.20)	0.43 (0.21 to 1.09)	0.480	0.023
	P_dTVuRRVn_	0.61 (0.59 to 0.66)	0.67 (0.59 to 0.77)	0.62 (0.56 to 0.76)	0.393	0.029
S_CY60_	P_RRV_ (e^−4^)	2.43 (1.15 to 3.52) ^O^	1.24 (0.89 to 2.12)	1.13 (0.71 to 1.95) ^Y^	**0.013 ***	0.133
	P_dTV_	63.30 (25.45 to 97.67)	141.55 (40.01 to 192.05)	99.11 (26.17 to 125.91)	0.307	0.036
	P_dTVuRRV_	32.34 (12.18 to 47.36)	72.16 (16.47 to 97.98)	48.60 (12.91 to 71.78)	0.340	0.033
	P_dTVrRRV_	30.55 (13.27 to 55.29)	67.71 (19.33 to 104.23)	42.66 (8.72 to 56.85)	0.301	0.037
	P_dTVuRRVn_	0.54 (0.50 to 0.57)	0.53 (0.50 to 0.55)	0.55 (0.51 to 0.58)	0.471	0.023
S_CY70_	P_RRV_ (e^−4^)	0.94 (0.45 to 1.67)	0.75 (0.33 to 1.03)	0.44 (0.24 to 0.91)	0.164	0.056
	P_dTV_	103.00 (57.80 to 222.93)	87.38 (66.51 to 172.59)	88.19 (49.36 to 213.62)	0.929	0.002
	P_dTVuRRV_	60.75 (29.50 to 122.93)	56.59 (33.15 to 95.72)	48.87 (26.85 to 117.75)	0.953	0.001
	P_dTVrRRV_	43.61 (27.82 to 105.93)	48.79 (24.36 to 76.87)	44.75 (15.30 to 93.96)	0.886	0.004
	P_dTVuRRVn_	0.56 (0.54 to 0.60)	0.55 (0.54 to 0.57)	0.55 (0.52 to 0.60)	0.555	0.018
S_CY80_	P_RRV_ (e^−4^)	0.32 (0.15 to 0.48)	0.19 (0.12 to 0.36)	0.20 (0.14 to 0.38)	0.345	0.033
	P_dTV_	151.85 (41.39 to 221.44)	96.18 (46.42 to 143.97)	79.23 (53.30 to 169.55)	0.789	0.007
	P_dTVuRRV_	85.69 (21.61 to 150.95)	58.99 (28.59 to 117.91)	53.67 (31.37 to 116.53)	0.898	0.003
	P_dTVrRRV_	53.54 (20.21 to 89.76)	31.17 (16.32 to 49.83)	31.73 (21.17 to 62.73)	0.506	0.021
	P_dTVuRRVn_	0.62 (0.58 to 0.69)	0.64 (0.61 to 0.70)	0.60 (0.56 to 0.68)	0.255	0.042
S_REC_	P_RRV_ (e^−4^)	7.09 (3.45 to 16.39)	10.71 (5.34 to 17.35) ^O^	5.16 (2.28 to 9.47) ^M^	**0.014 ***	0.130
	P_dTV_	2.46 (1.42 to 6.59)	2.83 (1.36 to 4.20)	4.92 (2.86 to 7.52)	0.086	0.075
	P_dTVuRRV_	1.25 (0.75 to 4.09)	1.30 (0.86 to 2.32)	2.66 (1.40 to 3.98)	0.067	0.083
	P_dTVrRRV_	1.27 (0.60 to 2.75)	1.43 (0.49 to 2.21)	2.23 (0.93 to 3.67)	0.257	0.042
	P_dTVuRRVn_	0.57 (0.54 to 0.64)	0.58 (0.50 to 0.62)	0.61 (0.54 to 0.64)	0.613	0.015

Values are expressed as median and interquartile range. Segments are based on the test phases: resting (S_REST_), cycling (S_CY_) and recovery (S_REC_). S_CY_ was divided in three stages at 60, 70 and 80% of estimated HR_max_, denoted as S_CY60_, S_CY70_ and S_CY80,_ respectively. P_RRV_ = LF oscillations for RR variability; P_dTV_ = LF oscillations for dT variability; P_dTVuRRV_ = P_dTV_ unrelated to P_RRV_; P_dTVrRRV_ = P_dTV_ related to P_RRV;_ P_dTVuRRVn_ = normalized P_dTVuRRV_. ***** = Significant differences between groups (*p* ≤ 0.05, Kruskal–Wallis test). ^Y^ = Different to Young adults; ^M^ = Different to Middle-aged adults; ^O^ = Different to Older adults.

**Table A2:** Comparison of P_RRV_, P_dTV_, P_dTVuRRV_, P_dTVrRRV_ and P_dTVuRRVn_ between BMI groups.

	Outcome	Non-Overweight (*n* = 38)	Overweight (*n* = 28)	*p*	ES
S_REST_	P_RRV_ (e^−4^)	16.93 (6.18 to 30.57)	10.09 (3.91 to 19.57)	0.143	0.180
	P_dTV_	1.10 (0.44 to 2.07)	1.60 (0.84 to 3.37)	**0.030 ***	0.267
	P_dTVuRRV_	0.57 (0.27 to 1.24)	0.75 (0.51 to 2.05)	0.078	0.217
	P_dTVrRRV_	0.46 (0.21 to 0.80)	0.75 (0.38 to 1.47)	**0.019 ***	0.289
	P_dTVuRRVn_	0.63 (0.60 to 0.71)	0.60 (0.56 to 0.73)	0.364	0.112
S_CY60_	P_RRV_ (e^−4^)	1.74 (1.01 to 2.95)	1.44 (0.73 to 2.33)	0.173	0.168
	P_dTV_	69.61 (27.79 to 165.01)	98.71 (23.81 to 176.69)	0.716	0.045
	P_dTVuRRV_	33.32 (12.88 to 74.32)	47.73 (12.13 to 90.98)	0.736	0.042
	P_dTVrRRV_	37.01 (14.92 to 78.70)	43.54 (8.67 to 89.04)	0.815	0.029
	P_dTVuRRVn_	0.55 (0.53 to 0.58)	0.51 (0.50 to 0.55)	**0.002 ***	0.388
S_CY70_	P_RRV_ (e^−4^)	0.72 (0.33 to 1.13)	0.54 (0.30 to 1.26)	0.645	0.057
	P_dTV_	99.71 (56.84 to 218.33)	87.78 (68.89 to 177.39)	0.887	0.018
	P_dTVuRRV_	53.14 (30.30 to 116.59)	56.33 (30.53 to 102.87)	0.979	0.003
	P_dTVrRRV_	43.61 (26.17 to 94.15)	46.17 (15.76 to 74.53)	0.640	0.057
	P_dTVuRRVn_	0.56 (0.54 to 0.60)	0.55 (0.51 to 0.59)	0.208	0.155
S_CY80_	P_RRV_ (e^−4^)	0.29 (0.14 to 0.41)	0.19 (0.13 to 0.46)	0.559	0.072
	P_dTV_	116.95 (51.73 to 235.29)	83.25 (51.81 to 150.11)	0.392	0.105
	P_dTVuRRV_	74.30 (31.29 to 148.63)	51.23 (25.08 to 114.92)	0.429	0.097
	P_dTVrRRV_	43.68 (19.03 to 93.20)	31.00 (19.81 to 55.13)	0.270	0.136
	P_dTVuRRVn_	0.64 (0.60 to 0.69)	0.61 (0.57 to 0.68)	0.254	0.141
S_REC_	P_RRV_ (e^−4^)	9.47 (6.05 to 18.15)	4.06 (2.17 to 8.44)	**<0.001 ***	0.436
	P_dTV_	2.76 (1.47 to 5.99)	3.62 (1.68 to 6.60)	0.517	0.080
	P_dTVuRRV_	1.27 (0.85 to 3.22)	2.16 (1.28 to 3.82)	0.259	0.139
	P_dTVrRRV_	1.49 (0.64 to 2.82)	1.59 (0.45 to 3.43)	0.825	0.027
	P_dTVuRRVn_	0.58 (0.53 to 0.64)	0.58 (0.53 to 0.63)	0.959	0.006

Values are expressed as median and interquartile range. Segments are based on the test phases: resting (S_REST_), cycling (S_CY_) and recovery (S_REC_). S_CY_ was divided in three stages at 60, 70 and 80% of estimated HRmax, denoted as S_CY60_, S_CY70_ and S_CY80_, respectively. P_RRV_ = LF oscillations for RR variability; P_dTV_ = LF oscillations for dT variability; P_dTVuRRV_ = P_dTV_ unrelated to P_RRV_; P_dTVrRRV_ = P_dTV_ related to P_RRV;_ P_dTVuRRVn_ = normalized P_dTVuRRV_. ES = Effect size. ***** = Significant differences between groups (*p* ≤ 0.05, Mann–Whitney *U*-test).

**Table A3:** Comparison of P_RRV_, P_dTV_, P_dTVuRRV_, P_dTVrRRV_ and P_dTVuRRVn_ between cardiorespiratory fitness level (PWC_80%_) groups.

	Outcome	Fit (*n* = 32)	Unfit (*n* = 34)	*p*	ES
S_REST_	P_RRV_ (e^−4^)	12.45 (5.61 to 24.60)	14.25 (6.12 to 28.66)	0.581	0.068
	P_dTV_	1.10 (0.43 to 2.22)	1.43 (0.80 to 2.70)	0.166	0.171
	P_dTVuRRV_	0.63 (0.27 to 1.38)	0.71 (0.43 to 1.24)	0.419	0.099
	P_dTVrRRV_	0.45 (0.23 to 0.80)	0.71 (0.33 to 1.44)	0.061	0.231
	P_dTVuRRVn_	0.65 (0.60 to 0.73)	0.61 (0.55 to 0.67)	0.059	0.232
S_CY60_	P_RRV_ (e^−4^)	1.23 (0.96 to 2.51)	1.82 (0.96 to 2.91)	0.441	0.095
	P_dTV_	74.15 (19.97 to 175.41)	92.39 (36.08 to 165.01)	0.635	0.058
	P_dTVuRRV_	35.28 (10.57 to 91.96)	44.43 (15.99 to 81.78)	0.663	0.054
	P_dTVrRRV_	36.43 (10.35 to 90.40)	48.12 (14.57 to 73.71)	0.599	0.065
	P_dTVuRRVn_	0.55 (0.52 to 0.57)	0.53 (0.50 to 0.55)	0.121	0.191
S_CY70_	P_RRV_ (e^−4^)	0.54 (0.32 to 1.03)	0.76 (0.35 to 1.32)	0.223	0.150
	P_dTV_	85.90 (42.46 to 208.57)	101.57 (70.22 to 175.55)	0.496	0.084
	P_dTVuRRV_	49.52 (21.85 to 121.80)	59.95 (34.25 to 91.91)	0.663	0.054
	P_dTVrRRV_	38.97 (16.83 to 86.07)	46.17 (31.58 to 78.83)	0.488	0.085
	P_dTVuRRVn_	0.56 (0.54 to 0.60)	0.55 (0.52 to 0.57)	0.166	0.171
S_CY80_	P_RRV_ (e^−4^)	0.21 (0.13 to 0.35)	0.26 (0.14 to 0.53)	0.133	0.185
	P_dTV_	130.30 (59.72 to 291.39)	83.25 (50.12 to 152.08)	0.281	0.133
	P_dTVuRRV_	82.98 (37.23 to 156.10)	49.39 (23.61 to 92.27)	0.178	0.166
	P_dTVrRRV_	42.06 (16.91 to 108.72)	31.45 (21.28 to 57.86)	0.635	0.058
	P_dTVuRRVn_	0.67 (0.61 to 0.70)	0.61 (0.58 to 0.64)	**0.016 ***	0.295
S_REC_	P_RRV_ (e^−4^)	8.39 (5.23 to 17.51)	5.22 (2.89 to 13.56)	0.063	0.229
	P_dTV_	3.55 (1.93 to 6.02)	2.86 (1.47 to 6.83)	0.710	0.046
	P_dTVuRRV_	2.03 (0.94 to 3.08)	1.36 (0.89 to 3.85)	0.672	0.052
	P_dTVrRRV_	1.81 (0.86 to 2.81)	1.44 (0.53 to 3.78)	0.691	0.049
	P_dTVuRRVn_	0.60 (0.54 to 0.65)	0.57 (0.53 to 0.62)	0.178	0.166

Values are expressed as median and interquartile range. Segments are based on the test phases: resting (S_REST_), cycling (S_CY_) and recovery (S_REC_). S_CY_ was divided in three stages at 60, 70 and 80% of estimated HR_max_, denoted as S_CY60_, S_CY70_ and S_CY80_, respectively. P_RRV_ = LF oscillations for RR variability; P_dTV_ = LF oscillations for dT variability; P_dTVuRRV_ = P_dTV_ unrelated to P_RRV_; P_dTVrRRV_ = P_dTV_ related to P_RRV;_ P_dTVuRRVn_ = normalized P_dTVuRRV_. ES = Effect size. ***** = Significant differences between groups (*p* ≤ 0.05, Mann–Whitney *U*-test).
